# Comparing conventional, biochemical and genotypic methods for accurate identification of *Klebsiella pneumoniae* in Sudan

**DOI:** 10.1099/acmi.0.000096

**Published:** 2020-02-10

**Authors:** Einas A. Osman, Nagwa El-Amin, Emad A. E. Adrees, Leena Al-Hassan, Maowia Mukhtar

**Affiliations:** ^1^​ Bioscience Research Institute, Ibn-Sina University, Aljerif West, Khartoum, Sudan; ^2^​ Microbiology Department, College of Medicine, Al-Qassim University, Al-Mulida, Saudi Arabia; ^3^​ Microbiology Department, College of Medicine, Alribat University Hospital, Khartoum, Sudan; ^4^​ Department of Global Health and Infection, Brighton and Sussex Medical School, Brighton BN1 9PX, UK

**Keywords:** Identification methods, *Klebsiella pneumoniae*, Sudan

## Abstract

*
Klebsiella pneumoniae* is recognized as one of the most important healthcare-associated pathogens worldwide due to its tendency to develop antibiotic resistance and cause fatal outcomes. Bacterial identification methods such as culture and biochemical tests are routinely used with limited accuracy in many low- and middle-income countries, including Sudan. The aim of this study was to test the accuracy of identification of *K. pneumoniae* in Khartoum, Sudan. Two hundred and fifty *K. pneumoniae* isolates were collected and identified using conventional phenotypic methods, biochemically using API 20E and genotypically by amplification of 16S−23S rDNA and sequencing of *rpoB*, *gapA* and *pgi*. Only 139 (55.6 %) of the isolates were confirmed as *K. pneumoniae* genotypically by PCR and 44.4 % were identified as non-* K. pneumoniae *. The results demonstrate that the identification panels used by the hospitals were inaccurately identifying *K. pneumonia* and led to overestimation of the prevalence of this organism. The current identification methods used in Khartoum hospitals are highly inaccurate, and therefore we recommend the use of a comprehensive biochemical panel or molecular methods, when possible, for accurate identification of *K. pneumoniae *.

## Introduction

*Klebsiella pneumoniae* has been medically recognized as one of the most important opportunistic pathogens, causing worldwide healthcare-associated infections (HAIs) such as pulmonary, urinary tract, blood and soft tissue infections [1]. Moreover, *K. pneumoniae* has become a clinically important micro-organism, particularly in the last two decades, due to its tendency to develop antibiotic resistance and cause fatal outcomes [2]. It is part of the ESKAPE organism group (* Enterococcus faecium *, *Staphylococcus aureus *, *Klebsiella pneumoniae *, *Acinetobacter baumanii*, *Pseudomonas aeruginosa* and *Enterobacter* species), which effectively ‘escape’ the effects of antibacterial drugs [3]. The use of limited routine methods (culture and conventional biochemical tests) to isolate pathogenic strains of *K. pneumoniae* may not be accurate due to the similarity of its biochemical reaction to that of other coliforms, leading to incorrect identification of the organism [4]. Unfortunately, these are the only methods of identification used in the hospital laboratories in Khartoum, Sudan, due to molecular techniques being unavailable as routine methods for identification and the expensive price of analytical profile index (API) kits. The burden of HAIs in general, and *K. pneumoniae* in particular, is not known in many low- and middle-income countries (LMICs) such as Sudan, due to the lack of adequate diagnostic and research infrastructure. The aim of this study was to determine the accuracy of identification of K. *pneumoniae* using the limited tests performed at the hospital laboratories.

## Methods

Two hundred and fifty isolates identified phenotypically as *K. pneumoniae* by the hospital laboratories were collected from four different hospitals (Rabat *n*=98, Souba *n*=52, Um Durman *n*=74 and Bahri *n*=26) in Khartoum state from April 2015 to December 2016. There is no specific algorithm for bacterial identification across most hospitals. Identification of *K. pneumoniae* from urine and wound swab samples in Khartoum hospitals is based on culture, colony morphology and Gram stain results. Blood and MacConkey agar is used for wound swab cultures, and blood and MacConkey agar or only CLED agar are used for urine samples. Colonies that are mucoid on blood agar, appear as Gram-negative rods under the light microscope after staining, and are lactose-fermenting mucoid colonies in MacConkey’s and CLED agar are identified as *K. pneumoniae* by the hospital laboratories. In other samples contributing to invasive infections [blood, cerebrospinal fluid (CSF) and pulmonary] a limited number of biochemical tests, such as indole tests, citrate tests, urease tests and Kligler iron agar (KIA) tests, are used subsequent to the identification by colony morphology and Gram staining (Fig. S1, available in the online version of this article). In the current study, API 20E/NE was used as an initial confirmatory test for 79 randomly selected samples . The isolates were cultured in MacConkey agar at 37 °C overnight, and processed as per the manufacturer’s instructions (File. S1). Further identification and confirmation of the all strains was carried out genotypically based on amplification of the 16S−23S rDNA internal transcribed spacer (ITS) of *K. pneumoniae* as previously described by Yin Liu *et al*. [5] Briefly, two pairs of *K. pneumoniae *-specific primers Pf (5ʹ-ATT TGA AGA GGT TGC AAA CGA T-3ʹ)/Pr1(5ʹ-TTC ACT CTG AAG TTT TCT TGT GTT C-3ʹ) and Pf/Pr2(5ʹ-CCG AAG ATG TTT CAC TTC TGA TT-3ʹ)5 were used for the PCR and analysed by gel electrophoresis. The PCR conditions were as follows: 1 µl template DNA (~10 ng, extracted using the Guanidine method) was amplified in a 25 µl containing 10 mM Tris–HCl (pH 8.3), 50 mM KCl, 1.5 mM MgCl2, 0.1 mM each of the four dNTPs, 1 unit *Taq* DNA polymerase and 1 µM of each primer. The cycling conditions were 10 min at 94 °C followed by 35 cycles of 30 s at 94 °C, 20 s at 57 °C and 20 s at 72 °C, followed by a 10 min hold at 72 °C. The *K. pneumoniae* isolates produced a 260 bp product with the Pf/Pr2 primer pair, in addition to a 130 bp product with the Pf/Pr1 primer pair. Other *Klebsiella* spp. (not *pneumoniae*) produced an amplicon with one primer pair but not with the other. Following identification with the 16S−23S rDNA ITS method, all *K. pneumoniae* isolates were confirmed by amplification and sequencing of the *rpoB* (beta-subunit of RNA polymerase), *gapA* (glyceraldehyde 3-phosphate dehydrogenase) and *pgi* (phosphoglucose isomerase), all of which are housekeeping genes in the *K. pneumoniae* genome [6]. The PCR conditions were similar to those used in the 16S−23S rDNA ITS methods, but with annealing temperature of 50 °C. The PCR products of the *rpoB*, *gapA* and *pgi* were purified using the Invitrogen PureLink PCR Purification kit and sequenced. Sequences were analysed using the blast database (https://blast.ncbi.nlm.nih.gov/Blast.cgi) to confirm that the sequences were specific to *K. pneumoniae *. The McNemar test was used to compare the specificity of the identification methods used by hospital laboratories.

## Results and Discussion

**Fig. 1.**
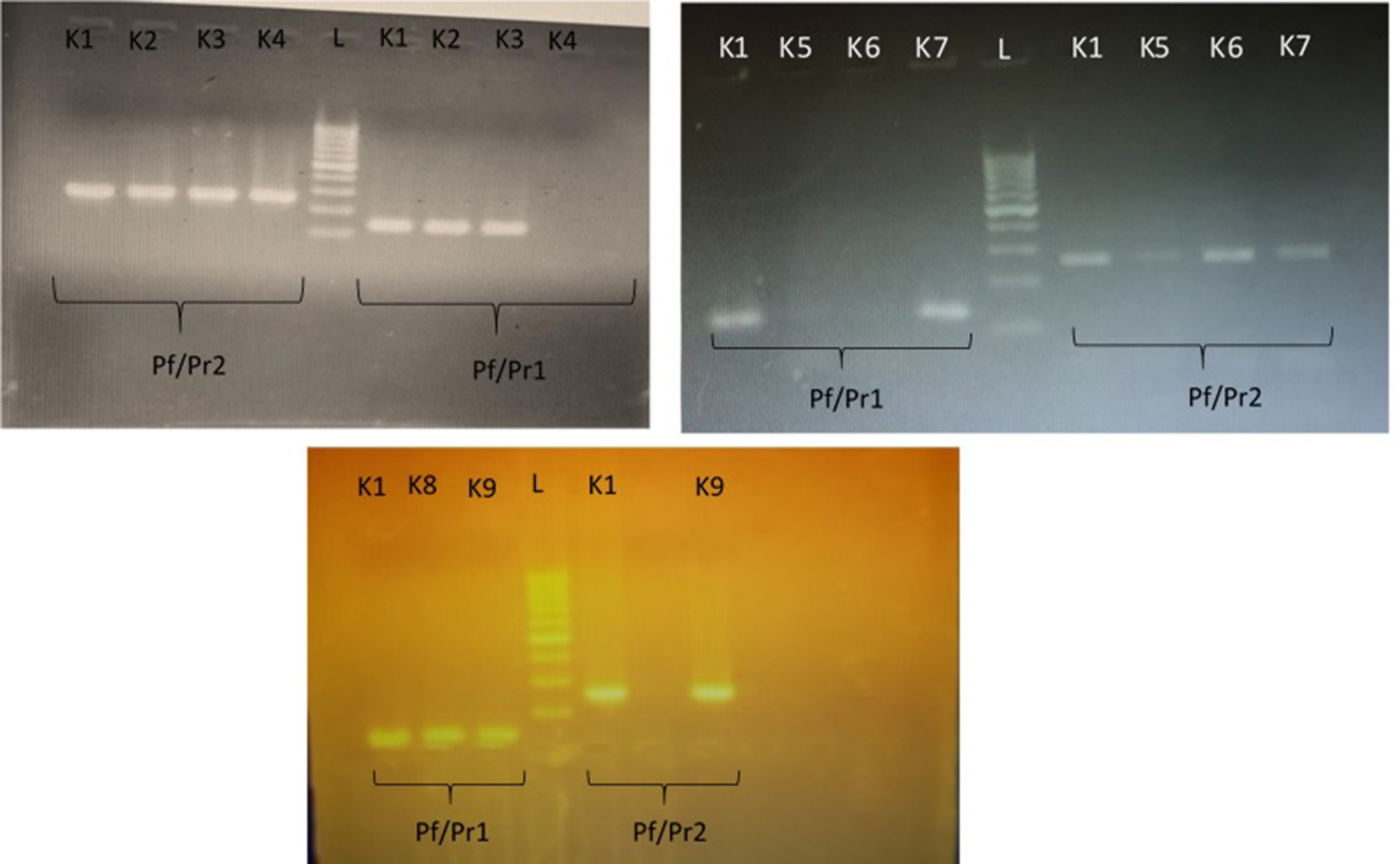
PCR amplification of the 16S−23S rDNA ITS of *K. pneumoniae *. Strain K1 was used as a positive control for all runs. *K. pneumoniae* isolates (K2, K3, K7 and K9) produced a band at 260 bp with the Pf/Pr2 primer pair, in addition to a 130 bp band with the Pf/Pr1 primer pair. Non-* K. pneumoniae* isolates (K4, K5 and K6) only produced a 260 bp band with primer pair Pf/Pr2 and failed to produce a band with Pf/Pr1, whereas K8 did not produce a band with Pf/Pr2, but did with Pf/Pr1. L, 100 bp ladder.

Of the 250 of Gram-negative isolates identified as *K. pneumoniae* by the clinical laboratory, 139 (55.6 %) were confirmed according to genotypic methods (16S−23S rDNA ITS followed by sequencing of housekeeping genes). The results of API 20E/NE for the 79 tested isolates showed that only 26 isolates were identified as *K. pneumoniae* (32.9%). The 26 isolates from the API-20NE were further confirmed using the 16S−23S rDNA ITS method and sequencing of the housekeeping genes. *K. pneumoniae* isolates produced 2=two bands with the primer pairs, as seen in Fig. 1 – a band at 260 bp with the Pf/Pr2 primer pair, in addition to a 130 bp band with the Pf/Pr1 primer pair. The remaining 111 (44.4 %) strains had been wrongly assigned as *K. pneumoniae* and only produced bands with one but not the other primer pair, as seen in Fig. 1. All 139 *K *. *pneumoniae* isolates underwent sequencing of the three housekeeping genes *rpoB*, *gapA* and *pgi*, confirming the accuracy of the 16S−23S rDNA ITS method.

**Fig. 2.**
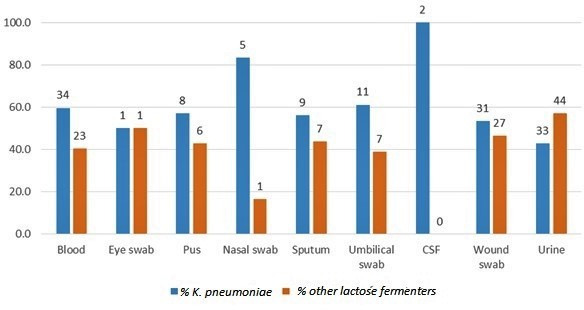
Samples from which *K. pneumoniae* was isolates. The blue bars indicate the number of isolates identified by the 16S−23S rDNA ITS, whereas the orange bars indicate the number of misidentified organisms.

Using the McNemar test we found that the specificity of the identification methods used by the hospitals is significantly lower than that of genotypic methods (McNemar chi-squared statistic with Yates correction of 0.5 is 110.002252, *P*-value is 0.000000).The bacteria studied were isolated from different clinical samples, as shown in Fig. 2 below. Our data show a high rate of misidentification for Gram-negative pathogenic organisms in Khartoum state, which does not give an accurate epidemiological picture, and may be contributing to wrong administration of antibiotics to patients. Identification of *K. pneumoniae* was only 100 % accurate in cerebrospinal fluid (CSF) samples, as these samples were subjected to different identification methods: culture in different kinds of media and using a panel of biochemical tests (such as citrate tests, urase tests, KIA tests, indole tests, MR tests, VP tests and motility tests). Conversely, accuracy was as low as 43 % for urine samples, where only colony morphology was used as the method of identification. Apart from CSF and nasal swabs, where 100 and 83%, respectively of *K. pneumoniae* were accurately identified by the laboratories, there was a large percentage of misidentification (39 –57 %) due to the difference in identification methods used for the different clinical samples (invasive vs non-invasive infections) (Table 1).

**Table 1.** 16S−23S rDNA *K. pneumoniae* identification versus phenotypic identification

	Blood	Eye swab	Pus	Nasal swab	Sputum	Umbilical swab	CSF	Wound swab	Urine
* K. pneumoniae *	34	1	8	5	9	11	2	31	33
Other lactose fermenters	23	1	6	1	7	7	None	27	44

The literature lacks sufficient evidence from Sudan, particularly on the burden of *K. pneumoniae* in healthcare settings. Hamdan *et al.* investigated the epidemiology of urinary tract infections from adult diabetic patients in Khartoum [7] and found that *K. pneumoniae* is the second most predominant cause of infections (after *E. coli*), causing 23 % of these infections. Here we studied *K. pneumoniae* isolates collected from various clinical specimens of patients in hospitals, which may explain the high prevalence at 55.6 % of all Gram-negative isolates collected as compared to the study that only studied urinary isolates.

Conventional biochemical tests, if not carefully selected, were shown by several studies to be inadequate in identifying *K. pneumoniae* [4, 8]. A study by Claus showed that the biochemical tests used to identify bacterial species may not be accurate and need to be tested to see if they are sufficient to discriminate between species [9]. On the other hand, genetic-based identification was shown to be highly accurate. Genes such as 16S−23S rRNA exhibit highly conserved polymorphism within *K. pneumoniae* clinical isolates, which make them a useful tool for the identification of *K. pneumoniae* isolates [10, 11]. Furthermore, it has been shown that using the 16S−23S rRNA gene ITS sequences to discriminate *Klebsiella* species and subspecies was feasible [11]. A study by Ahmed *et al.* (2015) stressed the need to use molecular biology techniques as essential diagnostic tools in microbiology laboratories [12], and the combination of microbiology and molecular biology led to high sensitivity and specificity. Low contamination levels and high speed have made molecular techniques appealing methods for the diagnosis of many infectious diseases. Molecular methods do, however, require costly equipment and expertise, and may therefore not be available in many LMICs due to lack of available funds.

The microbiological identification methods currently used in hospitals in Khartoum are highly inaccurate and no specific algorithm is used for bacterial identification. To avoid misidentification, we recommend that Khartoum state hospitals review and improve their routine identification methodology for pathogenic organisms to exhibit correct and accurate results. We recommend using molecular identification where possible to obtain the greatest accuracy. However, given the current limitations present in most hospitals (lack of infrastructure, facilities and funding), we would alternatively suggest including an appropriate biochemical testing panel to limit misidentification. The abbreviated algorithm for presumptive identification of *Enterobacteriaceae* tested by Ng *et al*. could be a good alternative [13].

## Supplementary Data

Supplementary material 1
