# Supplementary material for: Comparing conventional, biochemical and genotypic methods for accurate identification of Klebsiella pneumoniae in Sudan
Source: Access Microbiol. 2020 Feb 10;2(3):acmi000096. doi: 10.1099/acmi.0.000096 (PMC7470312; doi:10.1099/acmi.0.000096)
Supplement: Supplementary material 1 [file acmi-2-096-s001.pdf]

Supplementary data – Materials and Method

Figure s1: Sample processing at the microbiology laboratories for *K. pneumoniae* identification.

Please note this is performed routinely in the hospitals and not part of the current research project

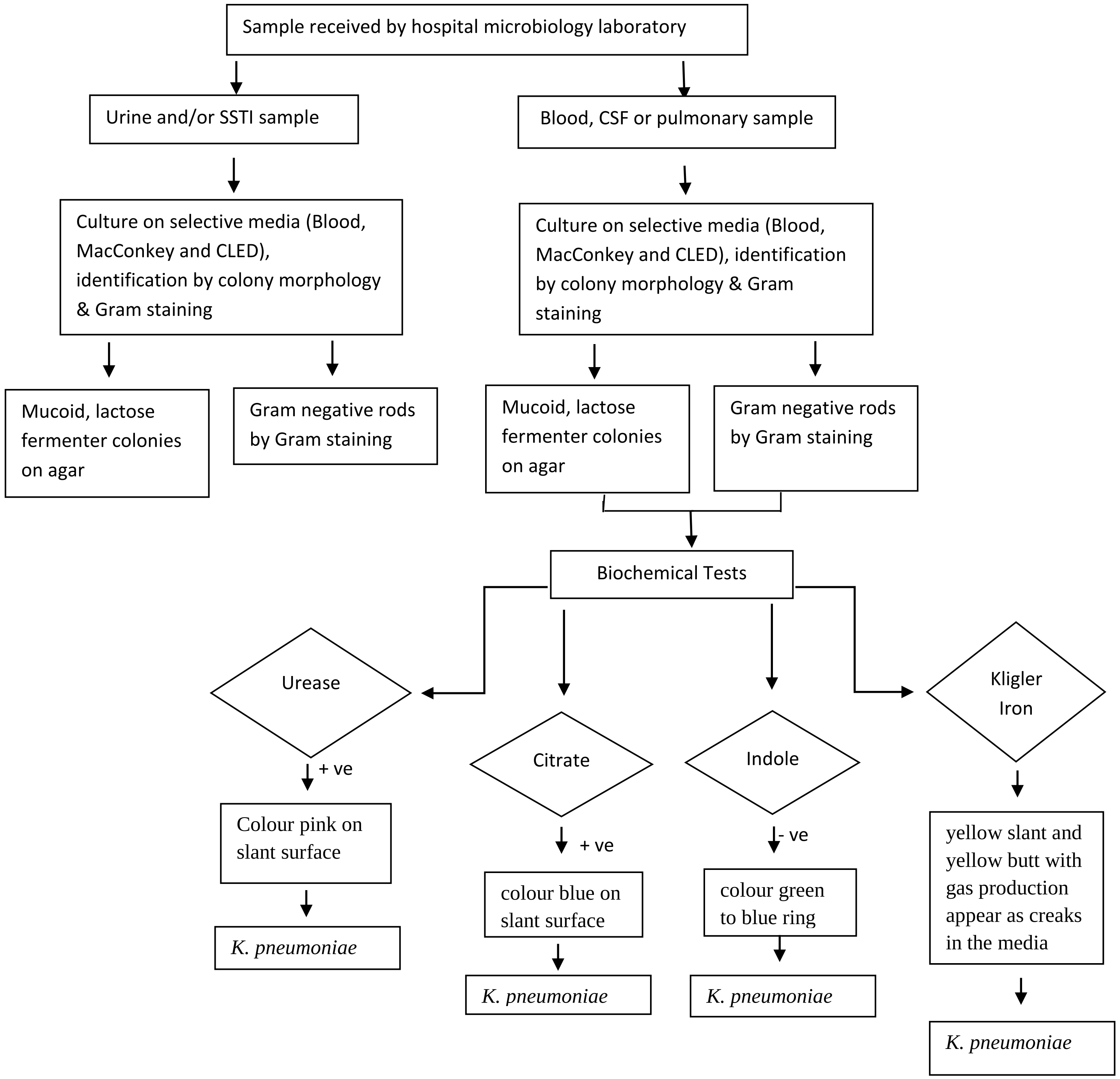

Figure s2: Diagram of all tests performed in the current study

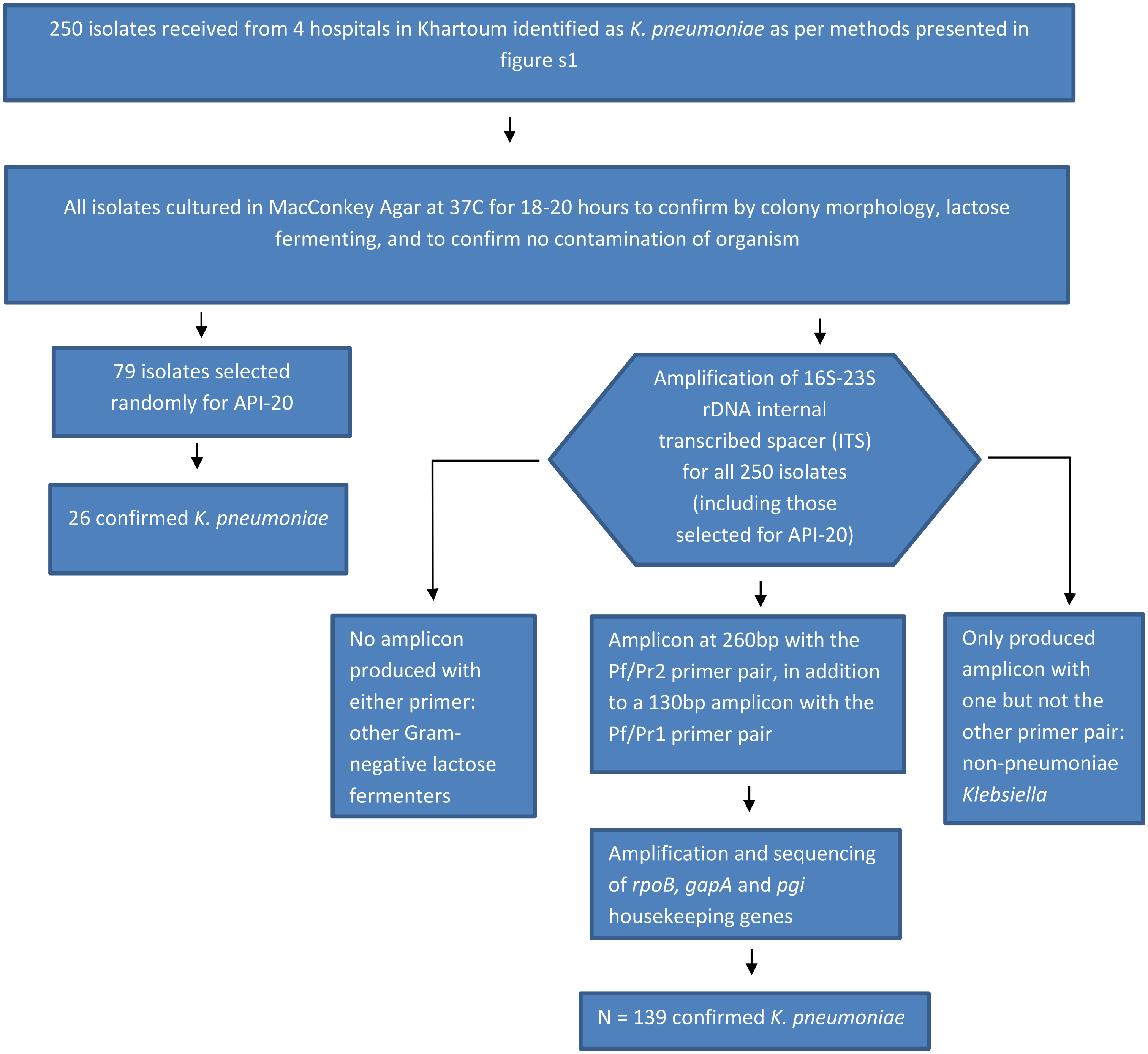

Method of API 20E/NE:

The isolates were cultured on MacConky agar at 37°C over-night, followed by suspension of the colonies in sterile normal saline. The solution was used to fill up the strip until the brim. Sterile oil was added to ODC, LDC, ADH, H_2S and urease test, and then the strips incubated over-night in 37°C. The following day one drop of Kovacs reagent was added for the Indole test , one drop of 40 % KOH (VP reagent 1) and one drop of VP Reagent 2 (α-Naphthol) were added for Voges-Proskauer test, and one drop of Ferric Chloride was added for the Tryptophan deaminase test. The results were read by using API Reading Scale (color chart) to determine a positive and negative result for each test, in the chart there are score for each positive test and each three reactions are added together at time to give a seven digital number. Then by using the apiwib (through inter the 7 digital numbers in to (https://apiweb.biomerieux.com) the organisms were identified.
